# Bicycle-inspired simple balance control method for quadruped robots in high-speed running

**DOI:** 10.3389/frobt.2024.1473628

**Published:** 2025-01-06

**Authors:** Shoei Hattori, Shura Suzuki, Akira Fukuhara, Takeshi Kano, Akio Ishiguro

**Affiliations:** ^1^ Division for Interdisciplinary Advanced Research and Education, Tohoku University, Sendai, Japan; ^2^ Research Institute of Electrical Communication, Tohoku University, Sendai, Japan; ^3^ Department of Electrical Engineering, Tohoku University, Sendai, Japan; ^4^ Japan Society for the Promotion Science, Tokyo, Japan; ^5^ School of Systems Information Science, Future University Hakodate, Hakodate, Japan

**Keywords:** quadruped robot, model-free dynamic balance control, high-speed running, high-speed turning, bicycle-inspired control

## Abstract

This paper explores the applicability of bicycle-inspired balance control in a quadruped robot model. Bicycles maintain stability and change direction by intuitively steering the handle, which induces yaw motion in the body frame and generates an inertial effect to support balance. Inspired by this balancing strategy, we implemented a similar mechanism in a quadruped robot model, introducing a yaw trunk joint analogous to a bicycle’s steering handle. Simulation results demonstrate that the proposed model achieves stable high-speed locomotion with robustness against external disturbances and maneuverability that allows directional changes with only slight speed reduction. These findings suggest that utilizing centrifugal force plays a critical role in agile locomotion, aligning with the movement strategies of cursorial animals. This study underscores the potential of bicycle balance control as an effective and straightforward control approach for enhancing the agility and stability of quadruped robots as well as potentially offering insights into animal motor control mechanisms for agile locomotion.

## 1 Introduction

Quadruped robots are increasingly being integrated into various industrial applications (Bjelonic et al., 2022). These robots have demonstrated remarkable versatility in tasks such as inspection, exploration, and patrolling. Their capability to select ground contact points enhances their adaptability on uneven and unstructured terrains, offering a notable advantage over wheeled and tracked robots. However, quadruped robots encounter distinct challenges in maneuverability compared to their wheeled and tracked counterparts (Ali et al., 2016). This limitation is primarily due to the intermittent ground contact of their legs and the greater shift in the center of mass during movement (Yongbin et al., 2022). Advancements in balance and turning control mechanisms are essential to further improve the mobility and agility of quadruped robots.

A hybrid approach that integrates features of wheeled systems has shown potential for enhancing the locomotion performance of quadruped robots. For instance, wheeled-legged robots use wheels as end effectors to traverse flat terrain with greater efficiency. The addition of actuated wheels allows legged robots to achieve efficient movement while preserving their ability to navigate rough and uneven terrains (Bjelonic et al., 2019; 2021; Klamt and Behnke, 2017). In contrast, passive wheels offer a solution to reduce energy consumption and robot weight (Hirose and Takeuchi, 1996; Endo and Hirose, 2012; Bellegarda et al., 2018). These studies underscore the considerable advantages of hybrid systems. However, incorporating wheel end effectors adds complexity to both the mechanical design and motor control required for effective legged locomotion.

This study introduces an alternative approach that leverages the advantages of both wheeled and legged systems. Specifically, we draw inspiration from the balance control mechanism of bicycles (Åström et al., 2005; Kooijman et al., 2011). Bicycles maintain stability and navigate direction by intuitively steering the handle, which induces a yaw motion in the body frame and generates centrifugal force. By utilizing this force, bicycles achieve smooth and stable directional changes, even at high speeds. Based on this balance control principle, we hypothesized that applying a steering mechanism for trunk control in quadruped robots could enhance their agility and mobility. In other words, instead of integrating features of wheeled systems into the end effectors, we incorporated them into the robot’s body frame.

This paper presents a quadruped robot model that incorporates balance strategies inspired by bicycle dynamics. Through simulation experiments, we investigated the feasibility of applying bicycle balance control principles to quadruped robots. The proposed model demonstrates dynamic stability during high-speed locomotion, even under external disturbances, along with a turning capability with only a slight speed reduction. Notably, parameter exploration shows that the model performs most effectively in walking and trotting gaits, likely due to the continuous ground contact assumed in bicycle-based balance control. These findings suggest that integrating a bicycle-like body frame structure can enhance stability and maneuverability in quadruped robots during high-speed movement.

The remainder of this paper is structured as follows. Section 2 provides an overview of the bicycle balance control mechanism and reviews related works. Section 3 describes the proposed quadruped robot model with a bicycle-inspired control strategy. Section 4 details the experimental setup and results, and Section 5 presents a discussion and the conclusions of this study.

## 2 Balance control mechanism of bicycle

This section describes the balance control mechanism of bicycles via a review of related works and a verification of a simple bicycle model. Bicycles change direction and turn smoothly and intuitively with the steering handle. When a bicycle moves forward and the rider steers the handle, the inertia of the bicycle resists changes from its straight motion. This results in an apparent centrifugal force, which helps the rider maintain balance and dynamic stability of the bicycle (Åström et al., 2005; Kooijman et al., 2011). For instance, once a rolling inclination happens, turning the steering to the ipsilateral direction helps correct the inclination. Since the centrifugal forces depend on the bicycle’s velocity and mechanical properties, it becomes unstable when the speed is insufficient (Åström et al., 2005). Utilizing centrifugal force through steering during high-speed movement is the balance control strategy for bicycles.

**FIGURE 1:**
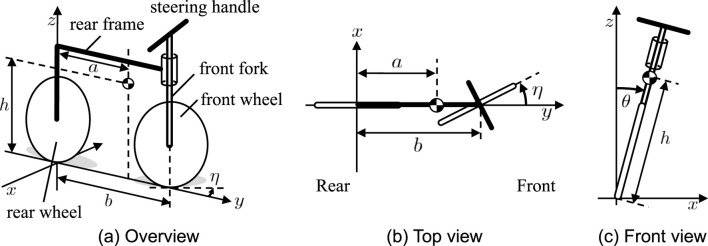
Simple bicycle model (Åström et al., 2005). **(A)** Overview, **(B)** top view and **(C)** front view. The steering angle is η , and the roll angle is θ .

Åström et al. (2005) investigated a simple bicycle model as shown in Figure 1 and demonstrated that a simple steering control can maintain the model posture. The model comprises four segments: the front wheel, rear wheel, front fork, and rear frame. The front fork and rear frame are connected perpendicularly. The steering is realized to rotate the front fork, which changes the wheel direction in the yaw direction, as shown in Figure 1B. The variable η is the steering angle. The total mass is concentrated at the center of mass. The equation of motion of the roll angle θ is as follows:

$$J\frac{d^{2}\theta }{d{\text{t}}^{2}}=mgh\theta +\frac{mv^{2}h}{b}\eta +\frac{Dv}{b}\frac{d\eta }{d\text{t}},$$ (1)

where J represents the inertia of the model around the roll axis, m denotes the total mass, g is the gravity constant, and h is the height of the center of mass; v is the velocity, the wheel base b is the length between the wheels, η is the steering angle, D is the inertial product. When the steering angle η is actively controlled in proportional to θ , as defined in Equation 2, and Equation 1 can be rewritten as Equations 3, 4,

η=−kθ, (2)

$$J\frac{d^{2}\theta }{d{\text{t}}^{2}}=mgh\theta +\frac{mv^{2}h}{b}\cdot -k\theta +\frac{Dv}{b}\cdot -k\frac{d\theta }{d\text{t}},$$ (3)

$$=\frac{mhk}{b}\left(\frac{bg}{k}-v^{2}\right)\theta -\frac{Dvk}{b}\frac{d\theta }{d\text{t}}.$$ (4)

When the velocity satisfies the condition $v^{2}>bg/k$ , the roll angle θ remains stable at zero. This indicates that the bicycle model with sufficient speed can stabilize its roll angle through the simple steering control. Furthermore, since this balance control approach does not rely on a wheeled structure, it has potential applications for quadruped robot designs as well.

## 3 Quadruped model

This section presents a quadruped robot model to investigate the applicability of the bicycle balance control mechanism, as described in Section 2. The balance control mechanism stabilizes posture by twisting the body frame through steering. Then, the proposed quadruped robot model implemented an actuated trunk DoF (Degree of Freedom) for steering and bicycle-inspired balance control. The following is a detailed description of the proposed model.

### 3.1 Mechanical structure

**FIGURE 2:**
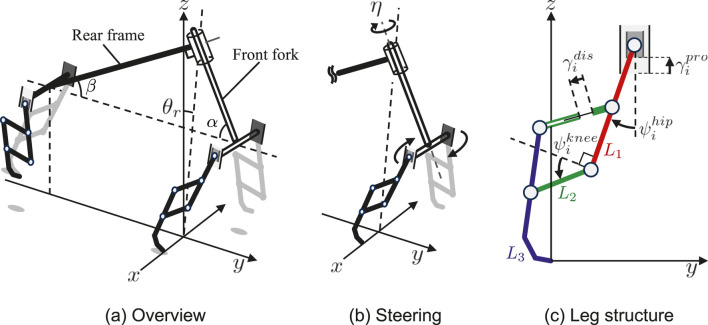
Mechanical structure of the quadruped model. **(A)** Overview **(B)** Steering mechanism that controls the trunk DoF in the yaw direction **(C)** Pantographic leg structure has four DoF: two actuated rotary joints at the hip and knee and two passive prismatic joints at the hip and pantograph.

Figure 2 shows the mechanical structure of the quadruped robot model. The robot model comprises a front fork, rear frame, and four legs with a pantograph mechanism. The legs are connected to the front fork and rear frame. The front fork and the rear frame are perpendicularly connected with a trunk joint serving as a steering joint. The trunk joint rotation makes the front legs rotate in the axis of the trunk joint, as shown in Figure 2B. The inclined front fork, adjusted by α , provides negative feedback from tilt to steering, resulting in self-stabilization, similar to the bicycle model described in (Åström et al., 2005).

The leg structure has a pantograph mechanism with four DoF, as shown in Figure 2C. The hip joint is an actuated rotary joint, which swings the leg in the anterior and posterior directions. The knee joint is an actuated rotary joint, which flexes and extends the leg with a pantograph mechanism. The two prismatic joints at the hip and the pantograph are passive spring dampers acting as suspensions.

### 3.2 Balance control

Drawing inspiration from the bicycle balance control, as described in Equation 2, we designed a balance control steering the trunk DoF angle η , as follows:

$$\bar{\eta }=-a\theta_{r}-\zeta$$ (5)

$$\tau =-k_{p}\left(\eta -\bar{\eta }\right)-k_{d}\dot{\eta }$$ (6)

where $\bar{\eta }$ denotes the target angle of the trunk joints, a is the weight of the feedback gain and $\theta_{r}$ is the roll angle of the rear frame, similar to the variable θ in Equation 4; ζ denotes the bias that controls the moving direction. The generated torque τ at the trunk joint is controlled by proportional-derivative (PD) control in response to the target angle $\bar{\eta }$ and angular velocity $\dot{\eta }$ of the trunk joint; $k_{p}$ and $k_{d}$ are the proportional and derivative gains, and $\dot{\eta }$ is the angular velocity of the trunk angle. The steering control is assumed to generate a centrifugal force that contributes to model balance.

### 3.3 Leg control

**FIGURE 3:**
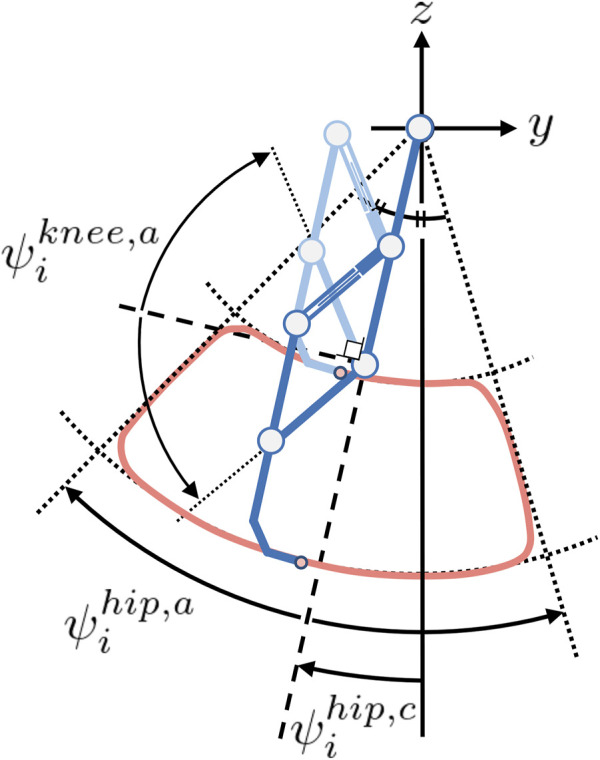
Leg trajectory. The deep and light blue legs represent the states of maximum and minimum extensions, respectively. The red line draws the leg trajectory. The roundness of the corners of the trajectory is determined by the constant ρ in Equation 8.

The leg actuators are controlled by PD control and make the foot tip draw the target foot trajectory, as shown in Figure 3. The trajectory was designed to emulate the cheetah’s high-speed locomotion pattern (Zhang et al., 2022). Key characteristics include the initiation of backward leg movement during the swing phase and the considerable clearance. The frequency is controlled by phase oscillators implemented in each leg. The oscillator phase $\phi_{i}$ determines the target angles of the hip joint ${\bar{\psi }}_{i}^{\mathrm{hip}}$ and the knee joint ${\bar{\psi }}_{i}^{\mathrm{hip}}$ as shown in Equations 7–9,

$${\bar{\psi }}_{i}^{\mathrm{hip}}=\psi_{i}^{\mathrm{hip,c}}+\psi_{i}^{\mathrm{hip,a}}\sin\phi_{i}$$ (7)

$${\bar{\psi }}_{i}^{\mathrm{knee}}=\left\{\begin{array}{cc} \psi_{i}^{\mathrm{knee,c}}-\psi_{i}^{\mathrm{knee,a}}\tanh\left(\rho \left(\phi_{i}-\frac{\pi }{2}\right)\right)\; & \left(0\leq \phi <\pi \right)\\ \psi_{i}^{\mathrm{knee,c}}+\psi_{i}^{\mathrm{knee,a}}\tanh\left(\rho \left(\phi_{i}-\frac{3\pi }{2}\right)\right)\; & \left(\pi \leq \phi <2\pi \right), \end{array}\right.$$ (8)

$${\dot{\phi }}_{i}=\omega ,$$ (9)

where $\psi_{i}^{\mathrm{hip,c}}$ is the offset angle of the hip joint and $\psi_{i}^{\mathrm{hip,a}}$ is the amplitude of the hip joint; $\psi_{i}^{\mathrm{knee,c}}$ is the offset angle of the knee joint and $\psi_{i}^{\mathrm{knee,a}}$ is the amplitude of the knee joint, and ρ is a positive constant; ω denotes the angular frequency of the oscillator $\phi_{i}$ . Here, the index i denotes the leg identifier (left fore: LF, right fore: RF, left hind: LH, and right hind: RH). The initiation of backward leg movement during swing phase is designed to set the offset angle of the hip joint $\psi_{i}^{\mathrm{hip,c}}$ , and the clearance from the ground is configured to determine the amplitude of the knee joint $\psi_{i}^{\mathrm{knee,a}}$ .

## 4 Simulation results

We conducted three experiments to evaluate the proposed model in the Open Dynamics Engine (Smith, 2005), a three-dimensional physical simulator. The first experiment investigated the running stability in multiple gaits, considering the differences between legged and wheeled locomotion. The second experiment investigated the robustness against disturbance. The third experiment investigated the turning performance.

All experiments were conducted on flat terrain, and the model parameters are listed in Tables 1, 2. The robot size and weight were determined somewhere between a typical quadruped robot and a bicycle, considering the effect of centrifugal forces contributing to stability. The robot size references that of a child’s bicycle and the weight references that of an actual quadruped robot (Unitree Go1^1^), and both are adjusted iteratively throughout the simulation experiments. Based on the mechanical parameters, the control parameters were determined as physically plausible values through a trial-and-error process. The initial conditions were as follows: the velocity v=0 [m/s], the steering angle η=0 [rad], the roll angle $\theta_{r}=0$ [rad], and the feet lifted 0.08 [m] from the ground. The remaining parameters are set for each experiment.

**TABLE 1:** Body and control parameters in simulation experiment.

Body parameters					
Total mass	11.0	[kg]	Leg mass	1.00	[kg]
Fore fork mass	5.00	[kg]	Rear frame mass	2.00	[kg]
Leg length	0.30	[m]	Trunk length	0.80	[m]
Shoulder/hip width	0.06	[m]	Leg link length $L_{1}$	0.20	[m]
Leg link length $L_{2}$	0.10	[m]	Leg link length $L_{3}$	0.24	[m]
Front fork angle α	60.0	[deg]	Rear frame angle β	30.0	[deg]
Control parameters					
a	1.00		$\psi_{i}^{\mathrm{hip,c}}$	20.0	[deg]
$\psi_{i}^{\mathrm{hip,a}}$ (forelimb)	35.0	[deg]	$\psi_{i}^{\mathrm{hip,a}}$ (hindlimb)	30.0	[deg]
$\psi_{i}^{\mathrm{knee,c}}$	0.00	[deg]	$\psi_{i}^{\mathrm{knee,a}}$	55.0	[deg]
ρ	3.0				

**TABLE 2:** Joint parameters in simulation experiment.

Rotary joint	P Gain [Nm/rad]	D gain [Nms/rad]	Max torque [Nm]
Trunk	2.0 $\times 10^{2}$	0.50	10.0
Hip	1.0 $\times 10^{4}$	30.0	50.0
Knee	1.0 $\times 10^{5}$	0.10	15.0

Linear joint	P Gain [N/m]	D gain [Ns/m]
Leg proximal	3.0 $\times 10^{3}$	100
Leg distal	3.4 $\times 10^{3}$	10.0

### 4.1 Locomotion sustainability with multiple gaits

Quadruped animals can move in various gaits (Alexander, 1984) unlike bicycles. The experiment investigated the gait variations of the robot model. The gait pattern is determined by the phase relationships between oscillators. We evaluated the locomotion performance for various gait patterns, which determine the left-right phase relationship $\Delta \phi_{LR}$ and the fore-hind phase relationship $\phi_{FH}$ derived as shown in Equations 10, 11,

$$\Delta \phi_{LR}=\phi_{LF}-\phi_{RF}=\phi_{LH}-\phi_{RH},$$ (10)

$$\Delta \phi_{FH}=\phi_{LF}-\phi_{LH}=\phi_{RF}-\phi_{RH}.$$ (11)

Here, when $\Delta \phi_{LR}=0$ [rad], the left fore leg and the right fore leg are inphase, $\phi_{LF}=\phi_{RF}$ . The hind legs are also the same. When $\Delta \phi_{FH}=\pi$ [rad], the legs of the ipsilateral side are antiphase, $\phi_{LF}=\phi_{LH}+\pi$ . We investigated the combination of $\Delta \phi_{LR}$ from 0 to π [rad] and $\Delta \phi_{FH}$ from 0 to π [rad]. The other experimental conditions are as follows: the phase angular velocity ω=28 [rad/s], the angle bias ζ=0 [deg], the experimental duration 15 [s], and ten trials for each phase relationship. In each trial, the initial phase $\phi_{LF}$ was set incrementally from 0 to 1.8 π in steps of 0.2 π . To evaluate the performance, we measured the locomotion sustainability. The gait pattern is regarded as sustainable when the running speed is 2.0 [m/s] or more at the end of the experiment.

**FIGURE 4:**
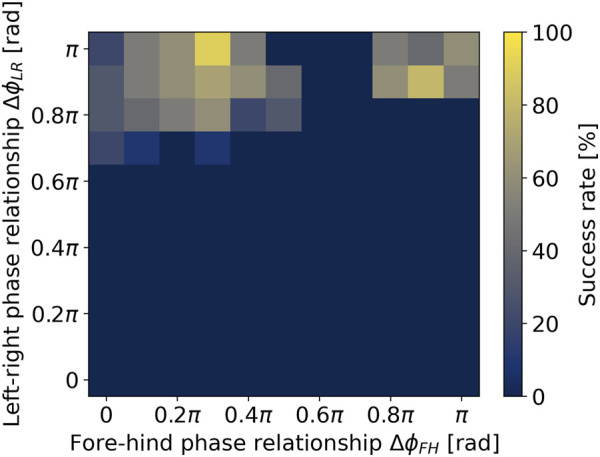
Locomotion sustainability with various gait patterns.

The simulation results are shown in Figure 4. The success rate represents the proportion of sustainable gait shown within ten trials for each parameter set. There are two parameter areas showing a higher success rate, although the robot cannot achieve stable running within most of the parameter set. The first area, $\Delta \phi_{LR}$ close to π [rad] and $\Delta \phi_{FH}$ close to 0.3π [rad], is a four-beat gait pattern similar to walking gait shown in slow locomotion of quadruped animal (Alexander, 1984). The second area, $\Delta \phi_{LR}$ close to π and $\Delta \phi_{FH}$ close to π is a two-beat gait pattern similar to trotting gait shown in intermediate-speed locomotion of quadruped animal. These results demonstrate that the robot model can achieve stable movement when following specific animal gait patterns. On the other hand, the robot cannot run at $\Delta \phi_{LR}$ close to zero, similar to galloping gait, which is the fastest gait of quadruped animals. This instability can be attributed to the characteristics of galloping, which involves a phase where both the left and right legs are off the ground. These characteristics are incompatible with the bicycle-inspired balance control, which assumes continuous grounding. Based on the results, we adopted the trot gait parameter set $\left(\Delta \phi_{LR}=\Delta \phi_{FH}=\pi \right)$ for the following experiments.

### 4.2 Robustness against disturbance

We conducted disturbance experiments that added the external force to the running robot model. The experimental duration is 7 [s], and the external force is 20 [N] in the x direction applied to the center of gravity of the rear frame from 4.0 to 4.5 [s]. The other experimental conditions are as follows: the phase angular velocity ω=28 [rad/s], the experimental duration 15 [s], the initial phase and phase relationship parameters $\left(\phi_{LF},\Delta \phi_{LR},\Delta \phi_{FH}\right)=\left(0,\pi ,\pi \right)$ [rad] that generate a trotting gait.

**FIGURE 5:**
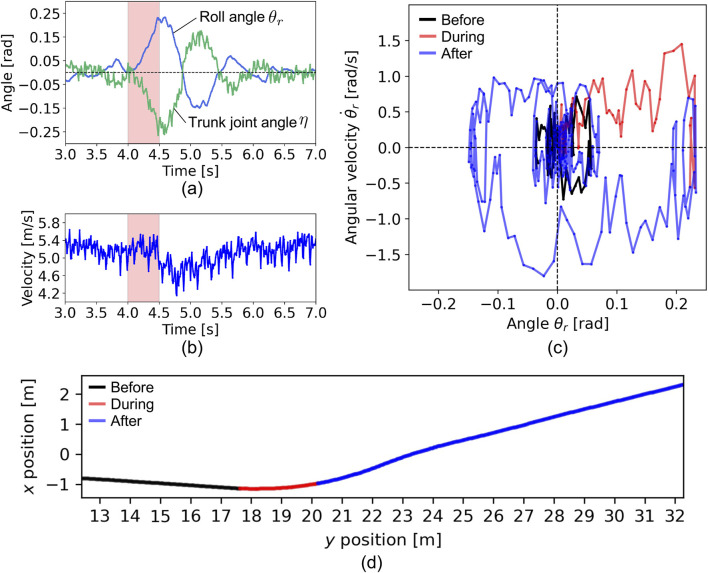
Disturbance experiment. The time evolution of **(A)** roll angle, trunk joint angle, and **(B)** velocity of the robot. The period (4.0–4.5 [s]) with the red color indicates the duration of the disturbance applied. **(C)** The relationship between the roll angle and angular velocity. The color of the lines indicates the time zone: black for before the disturbance, red for during, and blue for after. **(D)** Running trajectory on xy plane.

We observed the resulting behavior, as shown in Figure 5 and Supplementary Video S1. Figure 5A shows the time evolution of the roll angle $\theta_{r}$ and the steering angle η . Due to the disturbance, the roll angle tilted to 0.23 [rad] at 4.5 [s] and recovered to the neutral angle at around 6.0 [s]. The steering angle was controlled in response to the roll angle following Equations 5 and 6. The time evolution of the velocity, as shown in Figure 5B, also presents the impact of disturbance that decreases the speed temporarily.

Figure 5C shows the relationship between the roll angle $\theta_{r}$ and the angular velocity ${\dot{\theta }}_{r}$ . The color of the lines indicates the time zones: black for before the disturbance, red for during, and blue for after. The trajectory after the disturbance converges to the same range as the trajectory before the disturbance. It indicated that the robot’s posture has recovered, and the robot shows robustness against the external force.

Figure 5D illustrates the robot’s running trajectory on the xy plane. The movement direction aligns with the steering angle as the disturbance is applied. These results show that the proposed robot model can recover its posture from the external forces by steering the trunk joint in response to the roll angle and steering the trunk joint changes the direction of movement.

Next, we investigated the relationship between the oscillator angular velocity ω , related to the locomotion speed, and the robustness against external force. The experimental parameters are the oscillator angular velocity ω ranging from 20 to 38 [rad/s] and the external forces ranging from 0 to 45 [N]. For each parameter set, ten trials were conducted with the initial phase and phase relationship parameters set to $\left(\phi_{LF},\Delta \phi_{LR},\Delta \phi_{FH}\right)=\left(\pi ,\pi ,\pi \right)$ . To evaluate robustness across various postures, an external force was applied for 0.5 s, with the application timing shifted in increments of 0.1 of the gait cycle period ( 2π/ω [s]), beginning at 4 [s]. To evaluate performance, we used locomotion sustainability, as described in Section 4.1, as the metric.

**FIGURE 6:**
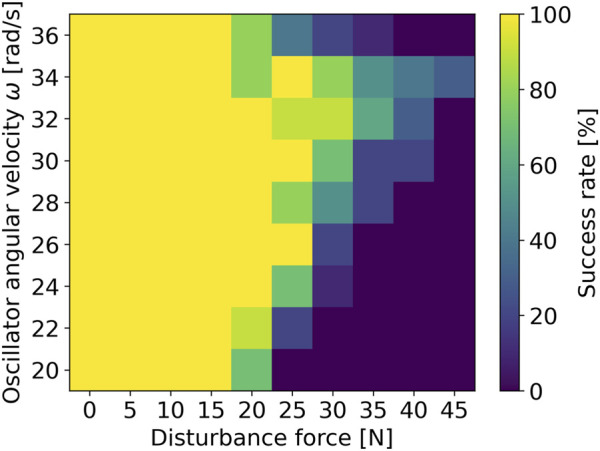
Relationship between the angular frequency of phase oscillator and robustness against disturbance.

The simulation results are shown in Figure 6. The success rate represents the proportion of sustainable gaits demonstrated after the disturbance, based on ten trials for each parameter set. There is a tendency that higher ω provide robustness against the greater external forces. When ω=34 [rad/s], the model can withstand a disturbance of 45 [N] in some trials. The reduced robustness against disturbances observed at ω=36 [rad/s] is likely due to insufficient steering feedback gain a for high-frequency locomotion. This property is consistent with the bicycle balance control, in which restoring force increases with speed, as described in Section 2.

### 4.3 Turning behavior

The steering handle is used to change the direction of movement of the bicycle, as well as to control balance. We conducted turning experiments to investigate the maneuverability of the proposed model. The turning behavior is generated by controlling the roll angel bias ζ as described in Equation 5. The experimental duration is 11 [s]. At 4 [s], ζ is set to 8.6 [deg]. When the robot achieves the target yaw angle, ζ is set to zero. The target yaw angle is π/2 [rad]. The other experimental conditions are as follows: The oscillator angular velocity ω=28 [rad/s], the initial oscillator phase $\left(\phi_{LF},\phi_{RF},\phi_{LH},\phi_{RH}\right)=\left(0,\pi ,\pi ,0\right)$ [rad].

**FIGURE 7:**
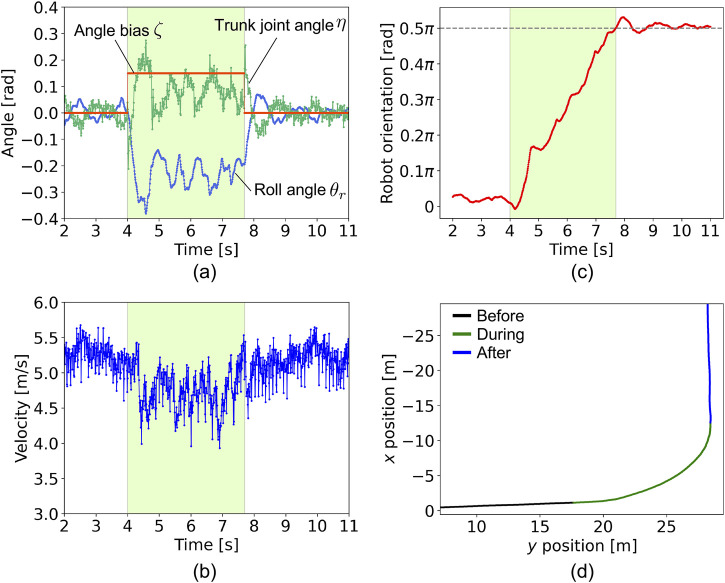
Turing experiment. The time evolutions of **(A)** roll angle $\theta_{r}$ , trunk joint angle η , roll angle bias ζ , and **(B)** velocity, **(C)** robot orientation representing the direction of movement. The duration of turning (4.0–7.7 [s]) is painted in green. **(D)** Running trajectory on xy plane.

We observed the turning behavior, as shown in Figure 7 and Supplementary Video S1. Figure 7A shows the time evolution of the roll angle $\theta_{r}$ , the trunk joint angle η , and the roll angle bias ζ . The angle bias ζ shifts to 8.6 [deg] from 4.0 until 7.7 [s], as shown in the green area. The trunk joint angle was controlled to achieve the angle bias ζ and make the roll angle tilt. During turning, the running speed slightly decreases, as shown in Figure 7B. Figure 7C shows the time evolution of the yaw angle of the robot, i.e., the direction of movements. The robot started to change the direction of movement at 4.0 [s] and reached the yaw angle of π/2 [rad] at 7.7 [s]. Subsequently, the yaw angle remained stable at π/2 [rad]. These movements are observed from the trajectory path, as shown in Figure 7D. The color of the lines indicates the time zone: black for before the turning, green for during, and blue for after. These results showed that the robot model changes the moving direction by simply changing the roll angle bias ζ .

**FIGURE 8:**
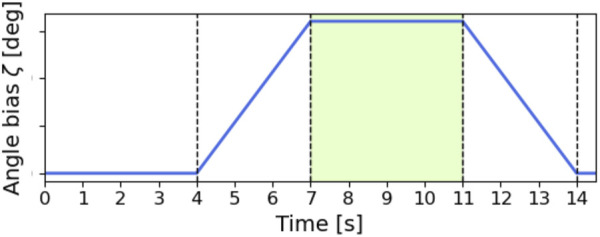
The time evolution of the roll angle bias ζ . The turning behavior was evaluated during the period (7.0–11.0 [s]) in the green area where ζ reaches its maximum value.

Next, we investigated the relationship between the turning performance and the parameter sets of the oscillator angular velocity ω and the roll angle bias ζ . The parameter sets are ω ranging from 18 to 36 and the maximum value of ζ ranging from 4 to 14. In each trial, the roll angle bias ζ was set to change linearly, as shown in Figure 8. The turning behavior was evaluated during 7–11 [s] where ζ reaches its maximum value. The turning radius is calculated from the ratio of the mean speed to the rotational velocity of the rear frame over the evaluation period. To evaluate performance, we used velocity, turn radius, and average roll angle. The experimental duration is 14 [s].

**FIGURE 9:**
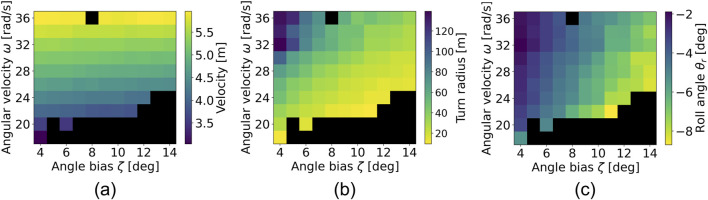
Color map of **(A)** velocity, **(B)** turn radius, and **(C)** roll angle. The black area indicates the trials in which falls occurred.

The simulation results are shown in Figure 9. Each color map shows velocity, turn radius, and average roll angle, respectively. The black area represents the trials in which falls occurred. The combination of lower ω and higher ζ tends to result in falls due to insufficient speed for sharp steering.

Figure 9A shows the velocity. The higher ω tends to run at a higher velocity. Figure 9B shows the turn radius. The higher ω tends to be a larger turn radius. It can be assumed that the higher velocity generates greater centrifugal forces to resist sharp turning. Besides, the larger angle bias ζ tends to be a smaller turn radius. Figure 9C shows that the angle bias ζ causes the roll angle to tilt, exhibiting a behavior similar to lean-in turning, which helps maintain high running speed, as observed in bicycle turning. The angle bias ζ affects the turn radius and not velocity, as shown in Figures 9A,B. The maximum rotational angular velocity reached 22.8 [deg/s], and the roll angle magnitude was 8.7 [deg] at the parameter set of ω = 22 [rad/s] and $\zeta_{\max}$ = 11 [deg]. The results show that the proposed model changes the moving direction by simply changing ζ and performs lean-in turning, maintaining a high running speed, as shown in bicycle turning.

## 5 Discussion

This study investigated the applicability of bicycle-inspired balance control in quadruped robot structures by introducing a yaw trunk joint as an alternative to a bicycle’s steering handle. The proposed model achieves stable high-speed locomotion (above 5 [m/s]) with robustness against external disturbances around 20 [N], along with maneuverability that allows for directional changes with only slight speed reduction, as shown in Figures 5–9. These results highlight the integration of bicycle balance control within a quadruped robot structure, demonstrating its feasibility for enhancing quadruped mobility. Unlike wheeled-legged robots (Bjelonic et al., 2022), which incorporate wheels into end effectors to enable both efficient locomotion and rough terrain adaptability, this study integrates wheeled-system features into the body frame. Whereas wheeled-legged designs typically require four additional degrees of freedom at the foot tips, our model requires only a single degree of freedom at the trunk, close to the center of mass, offering simpler implementation. This approach underscores the effectiveness of drawing inspiration from wheeled systems to improve quadruped agility without needing actual wheels.

Using centrifugal force through trunk movements for balance and turning aligns with locomotion strategies observed in quadruped animals (Kuznetsov et al., 2017; Wilson et al., 2013). Although previous studies introduced trunk joints in quadruped robots to enhance turning (Weinmeister et al., 2015; Wei et al., 2019; Lian et al., 2023; Wang et al., 2024), they did not account for the centrifugal effects that animals experience during sharp turns, nor did they demonstrate lean-in maneuvers. By explicitly demonstrating how centrifugal force aids balance control, this study contributes a novel insight into quadruped locomotion dynamics. Further exploration using the proposed model may enhance our understanding of animal high-speed motor control.

The proposed model is most effective in gaits resembling walking and trotting (Figure 4), where one front and one hind leg consistently maintain ground contact, similar to a bicycle’s continuous wheel-ground contact. However, the model struggles to achieve stable running in gaits like bounding and galloping, where the left-right phase relationship is closer to zero, typical of the fastest animal locomotion (Alexander, 1984). This limitation suggests that the model may only support a subset of animal gait patterns. Future work will develop additional control methods to enable gait patterns, including bounding and galloping, improving mobility and potentially contributing to our understanding of animal motor control.

Simulation results (Figures 5–9) indicate that centrifugal force contributes to both robustness against external forces and enhanced maneuverability. This approach, emphasizing the inherent dynamics of the model, is termed dynamics-based control (Osuka, 2001) in contrast to model-based control. For instance, passive dynamic walkers (McGeer, 1990) achieve natural gait using gravity alone, stabilized by implicit feedback structures derived from mechanical system (Sugimoto and Osuka, 2005). Our model similarly uses centrifugal force to simplify balance control, enhancing robustness without explicitly controlling roll posture. While conventional dynamics-based control relies on gravity, our approach demonstrates that centrifugal force can form the basis of a dynamics-based control strategy, providing insights into universal principles for dynamics-based control design in non-linear systems.

Future work should focus on further verification and investigation of the proposed model. First, conducting a theoretical analysis using Lyapunov methods, as well as comparisons with model-based and learning-based approaches, could help clarify the advantages of our model and establish a methodology for designing parameter sets, such as the roll angle bias ζ , for effective turning. Second, the model’s performance should be validated across various gait patterns, assessing its robustness against handling noise, external disturbances, and diverse terrain conditions. Additionally, exploring different body configurations will further demonstrate the applicability of the proposed model. Third, real-world validation is crucial for confirming the applicability and effectiveness of the proposed controller.

## Data Availability

The original contributions presented in the study are included in the article/Supplementary Material, further inquiries can be directed to the corresponding author.

## Footnotes

1
https://www.unitree.com/go1
